# Multichannel DenseNet Architecture for Classification of Mammographic Breast Density for Breast Cancer Detection

**DOI:** 10.3389/fpubh.2022.885212

**Published:** 2022-04-25

**Authors:** Shivaji D. Pawar, Kamal K. Sharma, Suhas G. Sapate, Geetanjali Y. Yadav, Roobaea Alroobaea, Sabah M. Alzahrani, Mustapha Hedabou

**Affiliations:** ^1^Department of Computer Science and Engineering, Lovely Professional University, Jalandhar, India; ^2^SIES Graduate School of Technology, Navi Mumbai, India; ^3^School of Electronics and Electrical Engineering, Lovely Professional University, Jalandhar, India; ^4^Department of Computer Science and Engineering, Annasaheb Dange College of Engineering and Technology, Sangli, India; ^5^NMC Royal Medical Centre Karama, Abu Dhabi, United Arab Emirates; ^6^Department Computer Science, College of Computers and Information Technology, Taif University, Taif, Saudi Arabia; ^7^School of Computer Science, Mohammed VI Polytechnic University, Ben Guerir, Morocco

**Keywords:** breast cancer, BIRADS Density Classification, DenseNet, deep learning, multichannel architecture, mammographic breast density

## Abstract

Percentage mammographic breast density (MBD) is one of the most notable biomarkers. It is assessed visually with the support of radiologists with the four qualitative Breast Imaging Reporting and Data System (BIRADS) categories. It is demanding for radiologists to differentiate between the two variably allocated BIRADS classes, namely, “BIRADS C and BIRADS D.” Recently, convolution neural networks have been found superior in classification tasks due to their ability to extract local features with shared weight architecture and space invariance characteristics. The proposed study intends to examine an artificial intelligence (AI)-based MBD classifier toward developing a latent computer-assisted tool for radiologists to distinguish the BIRADS class in modern clinical progress. This article proposes a multichannel DenseNet architecture for MBD classification. The proposed architecture consists of four-channel DenseNet transfer learning architecture to extract significant features from a single patient's two a mediolateral oblique (MLO) and two craniocaudal (CC) views of digital mammograms. The performance of the proposed classifier is evaluated using 200 cases consisting of 800 digital mammograms of the different BIRADS density classes with validated density ground truth. The classifier's performance is assessed with quantitative metrics such as precision, responsiveness, specificity, and the area under the curve (AUC). The concluding preliminary outcomes reveal that this intended multichannel model has delivered good performance with an accuracy of 96.67% during training and 90.06% during testing and an average AUC of 0.9625. Obtained results are also validated qualitatively with the help of a radiologist expert in the field of MBD. Proposed architecture achieved state-of-the-art results with a fewer number of images and with less computation power.

## Introduction

**Figure 1:**
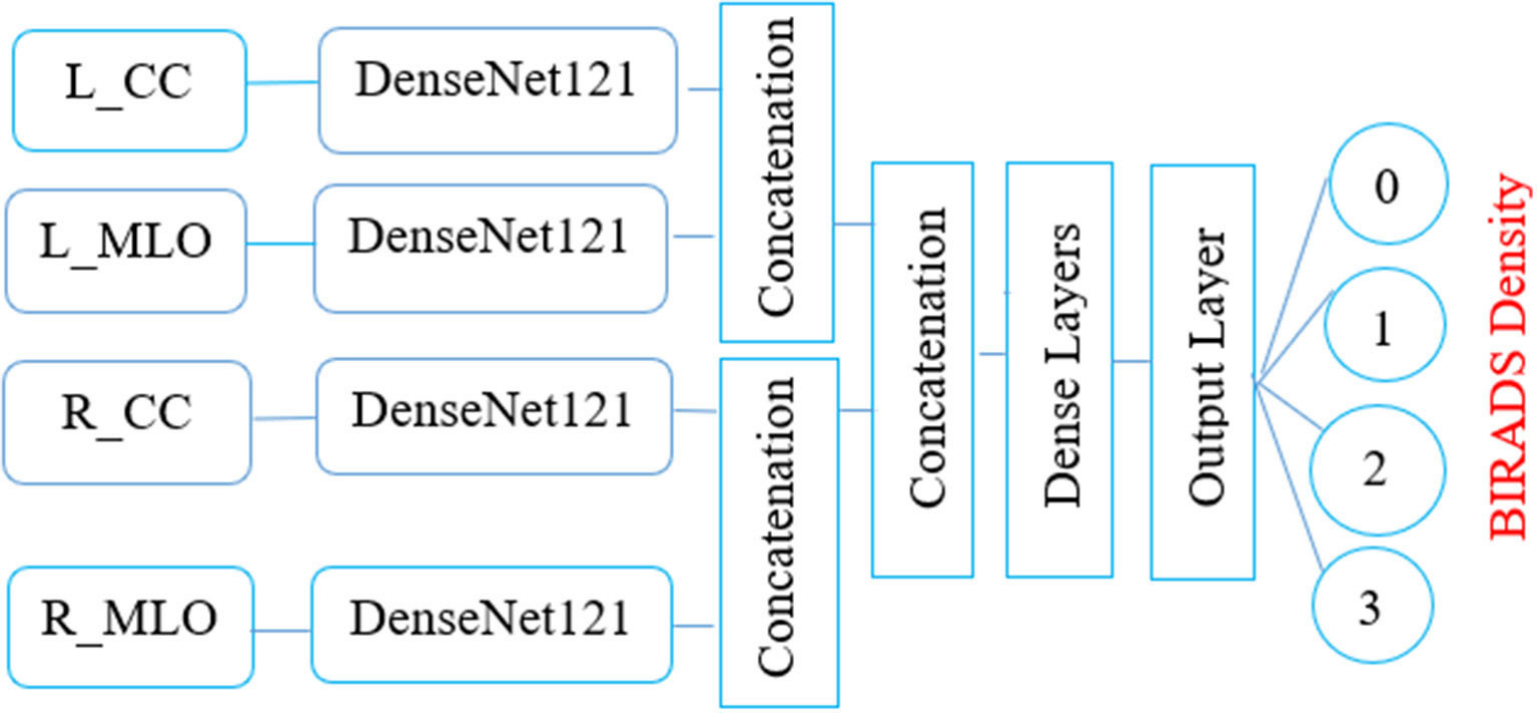
The proposed multichannel architecture for mammographic breast density classification.

Breast cancer has its reputation as a deadly disease and it is the second most frequent event of departure for the women community in society. It is frequently diagnosed in women with 12.3% compared to the average population (1–4). The incidence of breast cancer is associated with many biomarkers such as calcifications, masses, mammographic breast density (MBD), and architectural distortion. Advanced apprehension and proactive strategy are the unique alternatives to protect the lives of breast cancer cases and regress their subconscious shock (5–8). MBD is an essential biomarker in interpreting digital mammograms and preparing a systematic mammography screening program. According to epidemiological investigations, females with highly dense breast tissue risk developing breast malignancy (9, 10). MBD is a radiographically visible density on the mammogram, consisting of lobular elements, ducts, and fibrous connective tissue compared to the lucent fatty tissue in the breast (11). The reports are prepared during the digital mammography screening program as per the American College of Radiology Breast Imaging Reporting and Data System (ACR BIRADS) catalog. This catalog was last modified in November 2015 (12, 13). As per the BIRADS, MBD gets classified into the four significant groups of mammographic breast density as class A, class B, class C, and class D, which reduces the sensitivity of digital mammography (14). Figure 1 depicts all the MBD BIRADS classes.

Different scientific studies have revealed that digital mammograms' sensitivity strongly depends on the density class of the breast tissue. In dense breasts, the sensitivity of mammograms is as low as 63%, while in the low-density breast, there is an exponential rise of 87%. Hence, patients with high-density breasts have to go for additional imaging such as tomosynthesis, ultrasound, or breast MR to enhance cancer detection chances (15). Researchers have proposed many semiautomatic and automatic approaches for measuring breast density from the last two decades. However, the assessment of mammographic breast density (MBD) is subjective, which expert radiologists do (16). Despite this labeling, due to poor interreader and intrareader reproducibility, MBD classification has many limitations (17).

For an automatic objective assessment of MBD classification, many study efforts have been in progress from the last few decades. The initial study focuses on image processing techniques such as area-based thresholding, region growing, and clustering algorithms. A significant step formulated was the emergence of the machine learning (ML) algorithms based on different extracted image topographies from the histogram, texture intensities, patterns, and image acquisition parameters. Nowadays, deep learning algorithms provide yet another leap forward in MBD classification. Deep learning algorithms can go substantially deeper and discover all the significant features from the image. Due to system architecture and hardware improvements, it is possible to train deep learning architecture intensely. This improvement makes deep learning architecture an excellent tool for medical image analysis. Many deep learning architectures such as LeNet, (visual geometry group) VGG19, highway networks, residual networks, and DenseNet are recorded in literature.

Nevertheless, before every deep learning network lets more intelligence, a new study intricacy happens, the “vanishing gradient problem.” DenseNet (dense convolutional network) provides unique insight to secure the best data flow between layers to solve the connectivity problem. This interface immediately combines all the layers, agreeing on characteristic map dimensions in feed-forward type; hence, the individual layer receives input from all the previous layers also transfers its distinct map to all the farther layers. Thus, the DenseNet concatenate feature map passes through all the subsequent layers instead of summarizing features such as ResNet. This concept includes L (L+1)/2 connections instead of L connections, identifying a dense connectivity pattern (18). The consequence of this connectivity guide, i.e., DenseNet architecture, provides the following advantages:

1. Effective solution for gradient vanishing.
2. Consolidation in characteristic distribution.
3. Provision of feature recycle.
4. Significant reduction in training parameters.
5. Easy to train and offers better parameter efficiency.

Due to these advantages, it is helpful to use this model without pretraining for medical image analysis.

**Figure 2:**
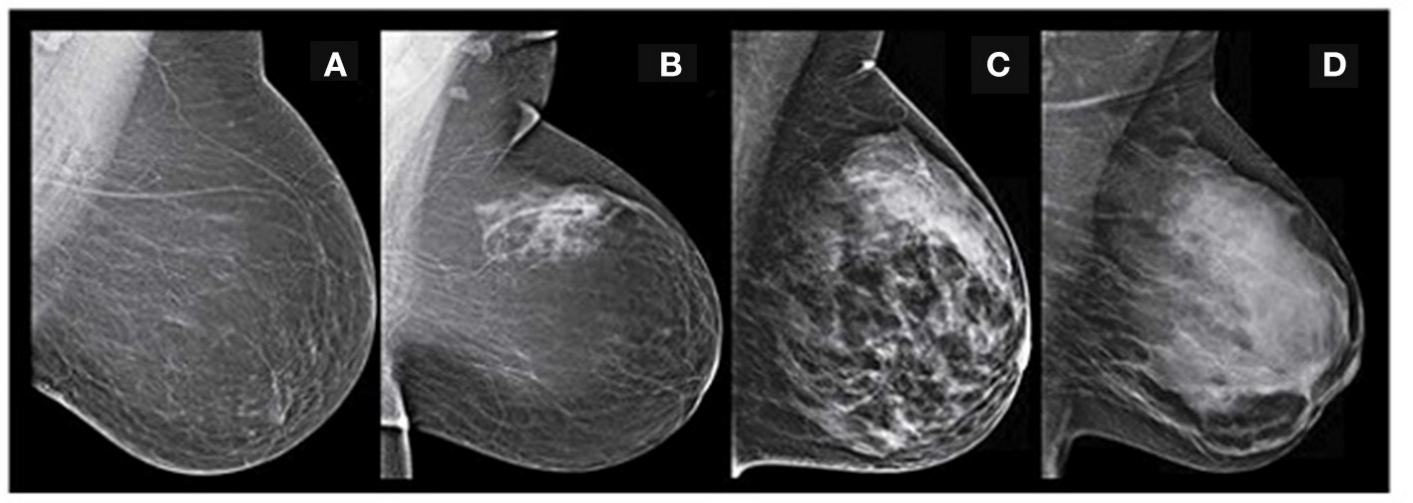
BIRADS classification-**(A)** fatty-class A **(B)** fat with some fibro glandular tissue -class B **(C)** heterogeneous dense-class C **(D)** extremely dense-class D (Image courtesy: Densebreast-info.org).

The elemental aspiration behind this study is to investigate the use of multichannel DenseNet architecture for MBD classification and analyze the proposed architecture compared with different existing methods. Figure 2 depicts the proposed multichannel architecture for MBD classification.

The key feature of this architecture is classifier performance that is evaluated with 800 digital mammograms among the diverse BIRADS density categories. Results are confirmed exclusively by expert radiologists and objectively with classification accuracy and the area under the curve (AUC). Various sections described in this article are as below. Related study section covers unique existing deep learning algorithms for MBD classification, Study dataset section presents the aspects of the source dataset, Proposed methodology section represents the proposed architecture, and Analysis of experiment and outcomes section introduces the empirical outcomes. Finally Discussion section discusses the future scope and Segment 7 concludes this study.

## Related Study

Mammographic breast density classification is a long-lasting study area due to more thought-provoking and challenging image preprocessing, segmentation, and classification tasks. Successful deep learning classifiers such as ResNet, VGGNet, and GoogLeNet give brand-new pathological imaging and investigation prospects. This section describes the remarkable of such existing methods used for MBD classification. In summation, these classifiers offer better results on different imaging modalities for image classification. Intrinsically, high interreader fluctuations are the prime problem in MBD assessment. To overwhelm this problem, Ciritsis et al. (19) suggested deep learning architecture with 11 convolutional layers, three fully connected layers, and an output SoftMax layer to distinguish breast density into two classes and four classes. Two expert radiologists marked density ground truth during this classification task. A combination of mediolateral oblique (MLO) and craniocaudal (CC) (left or right) views (20,578) digital mammograms were used to train the proposed model. During the training, to enhance the model's performance and desist overfitting, the convolutional layers are zero-padded and used a dropout rate of 50%. Batch size and the highest number of epochs used during training are 40 and 120, respectively. Input data segmented as 70% for training and 30% for validation. This model is validated independently on CC and MLO views for two-class classification and achieved an overall classification accuracy of 89.9 and 86.6%.

Predominantly, fat appears darker than fibroglandular tissue in MBD assessment. Hence, the pixel intensity of the histogram acts as an essential feature for classifier training. Wu et al. (20) suggested this technique for MBD classification. In this method, the SoftMax regression classifier is used to learn pixel intensity histograms. Four regular views [left CC (LCC), right CC (RCC), left MLO (LMLO), and right MLO (RMLO)] of 2,00,000 screening images were given individually to train the prototype. The proposed architecture is deep and consists of 100 hidden units between input and output layers. All the hidden layers use Rectified Linear Unit (ReLU) as an activation unit. This model is tested for two- and four-class classification and achieved 81.1 and 82.5% classification accuracy. Thus, this model provides moderate classification accuracy despite an extensive dataset. Lizzi et al. (21) suggested a residual convolutional network for MBD classification. The recommended design consists of 41 convolutional layers formed in residual blocks with 2 million parameters. The first block of this architecture consists of a convolutional layer, a batch normalization layer, a leaky ReLU as an activation function, and a two-dimensional (2D) max pooling. A series of four sections consisting of three residual modules uses the output features of the input block. Leaky ReLU with α = 0.2 activation functions was used to train the architecture. Categorical cross-entropy as a loss function validates the performance of the model. Maximum accuracy with four-class classification is 78% and the two-class accuracy is 89.4%.

In a comparative study for evaluating the performance of deep learning and transfer learning on a similar dataset, Lee et al. (17) proposed both the approaches on 22,000 mammographic images. In this approach, expert radiologists marked density ground truth for the input images. Initially, the model's training starts with 500 images with the AUC of 0.942 and the final value of the AUC is 0.9882 reported on the whole dataset. Then, the proposed model is cross-validated with the transfer learning (ImageNet) application and obtained the overall AUC of 0.9857. Thus, this study shows that both the deep learning and transfer learning applications provide almost identical results on the equivalent dataset. The requirement of a larger dataset for training is an essential need of deep learning architecture. Shi et al. (22) proposed optimized lightweight deep learning architecture to optimize deep learning performance on smaller datasets. This architecture combines three convolutional neural networks (CNNs), one dense layer, and an output layer with the SoftMax function. Data augmentation is done with additional image processing to increase the numbers in the dataset. This architecture was trained and tested on the 322 mini-Mammographic Image Analysis Society (MIAS) dataset. This architecture provides overall accuracy of 83.6% on four-class classification. The main limitation of lightweight architecture on smaller datasets is the low stability of the network, which may occasionally cause large and significant variations in the accuracy. Data augmentation can enlarge the dataset in this method, but it is still challenging to get well-trained convolutional layers due to little diversity between the original and generated datasets. In the literature, there are two ways that are recorded to enhance the model performance with a smaller dataset, which are generative adversarial network (GAN) and another is transfer learning (23).

Recently, different researchers proposed the concept of transfer learning on a smaller dataset. For example, Kaiser et al. (24) proposed new architecture that can take all the four views of single patients to classify MBD into two-class classification (dense and nondense). For this purpose, the author proposed four-channel VGGNet architecture to extract all the features with average global pooling from input mammograms. Before the classification layer, to concatenate all the input layer features, two dense layers are used. Then, the proposed model is trained with 5-fold cross-validation and recorded 88% classification accuracy with the AUC of 0.954. Finally, subjective assessment is done with a panel of 32 radiologists to compare interobserver variability. In this approach, interobserver variability for breast density assessment is observed even high in two-class classification. Thus, the automated processes for MBD can help to minimize interobserver variability. Despite different automated approaches, MBD assessment is subjective and consists of intra- and interobserver variations. Objective evaluation of other commercially viable methods consists of mixed evaluation results. Another fundamental limitation is that most of existing deep learning methods need a higher dataset and validated density ground truth; hence, data acquisition becomes difficult for researchers. In addition, mammographic images are vendor specific, making deep learning more robust; training the deep learning model through different vendor-specific samples is required, another bottleneck in MBD classification. All the limitations mentioned above result in moderate objective MBD classification accuracy.

The primary motivation behind this study is to investigate the transfer learning application of DenseNet architecture toward enhancing the classification accuracy of MBD. A significant contribution is the design and development of multichannel architecture to utilize four mammographic views of a single patient.

## Study Dataset

The intended study utilizes the openly available dataset from digital database for screening mammography (DDSM) (25), consisting of 2,620 samples of various classes labeled as benign, normal, and malignant with confirmed pathogeny data. The aimed algorithm uses 200 Right-MLO, 200 Left_MLO, 200 R_CC, and 200 L_CC views. A total of 800 mammograms are used for training and testing purposes. The ground truth of each class is labeled with the help of specialist radiologists team into four classes as 0, 1, 2, and 3 as a four MBD classes. All the density groups consist of 200 cases of different images (MLO, LMO, R_CC, and L_CC). Table 1 presents the details of the ground truth input dataset used in this proposed study.

**Table 1:** Input dataset used for testing and validation of proposed algorithm.

**BI_RADS Density Class**	**Number of images**
Class-A	200
Class-B	200
Class-C	200
Class-D	200
Total	800

## Proposed Methodology

This segment explains the proposed MBD classification technique and divided into three subsections as presented subsequently.

### Segmentation of Pectoral Muscle

**Figure 3:**
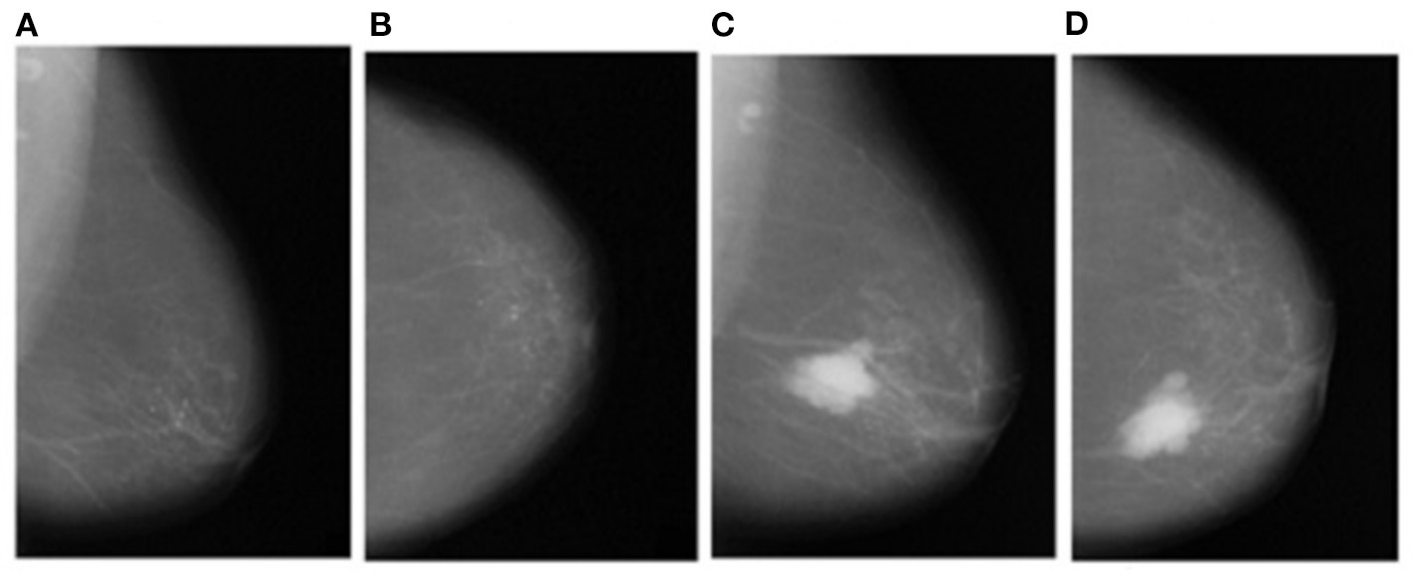
Input raw images- **(A)** Left_MLO **(B)** Left_CC **(C)** Right_MLO **(D)** Right_CC.

**Figure 4:**
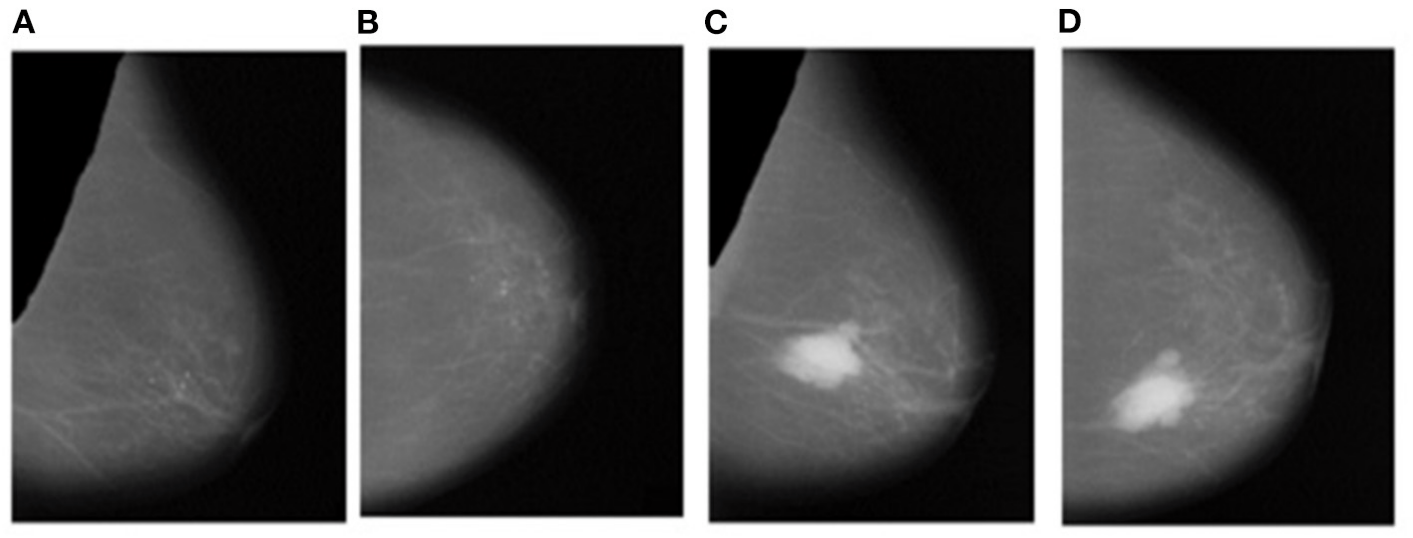
Output Images after segmentation and cropping - **(A)** Left_MLO **(B)** Left_CC **(C)** Right_MLO **(D)** Right_CC.

Instead of using raw images for models' training, the proposed method preprocesses both the LMO and MLO views. Input mammographic images consist of high-intensity marks of artifacts and tags and pectoral muscle. Those fields in the breast region can reduce the MBD classification accuracy (26, 27). The proposed method uses the depth-first search algorithm, our previous study (28), to remove pectoral muscle, artifacts, and tags from all the MLO and LMO views. This preprocessing study helps the model to classify all the BIRADS classes correctly with less input images. Depth-first search (DFS) algorithm identify all the unwanted high-intensity areas (artifacts and pectoral muscle) of MLO and CC views and removes them, which further help the model to make the correct decision. Figures 3, 4 depict the input and output images after preprocessing.

### Contrast Enhancement

Mammographic breast density classification is a function of the density of fibroglandular tissues inside the breast. Contrast enhancement helps to improve the visibility of fibroglandular tissues. Subsequently, it helps to improve the classification accuracy of deep learning models. In literature, many contrast enhancement methods are recorded to enhance the quality of medical images. These methods are “histogram equalization (HE),” “adaptive histogram equalization (AHE),” and “wavelet transform (WT) coefficients” (29–31). But, these documented practices take ample processing time and are less effective in noise reduction. Another recorded method is the “unsharp masking (USM) method,” which enhances the local contrast inside the image by limiting the global contrast. Still, this technique creates artifacts in the image. Due to this, the image looks artificial; therefore, it is not fitting for the enrichment of the medical images (32–34).

In contrast to gain agreement for the high-frequency element of the image, which is the basic principle behind “adaptive contrast enhancement (ACE),” it consists of limitation in terms of high processing time (32–38). In MBD classification, local details are more important than global features and reduce processing time; the proposed architecture uses the contrast limited adaptive histogram equalization (CLAHE) algorithm for limited contrast enrichment of fibroglandular tissues (39–41). The first merit of this method helps to minimize the edge shadowing effect and noise produced in homogeneous input digital mammograms. Second, small images known as tiles are used instead of the entire image to perform the CLAHE operation. Hence, contrast enhancement of each tile histogram matches with exponential distribution or Rayleigh distribution. Moreover, to overcome artificial-induced borders, neighboring tiles are connected by bilinear insertion. This advanced CLAHE technique is outlined under:

1. Initially, all the input mammograms are divided into 8 × 8 non-overlapping contextual fields of equal sizes and later a histogram of various contextual regions is calculated.
2. The clip limit (β) is the threshold parameter used to alter the contrast of the image, which is calculated by Equation (1).

(1) $$\begin{array}{l} \beta =\frac{M\;\times \;N}{L}\left(1+\frac{\propto }{100\;}\left(S_{\max}-1\right)\right) \end{array}$$

Where β is the clip termination, calculated as eight by several experiments, M × N is the number of pixels in each field, L is the number of gray scales, and α is a clip factor (0–100). It is the highest allowable slope, which is set to be 4 for this analysis.

3. Each histogram is aligned, so that its maximum does not surpass further than the clip boundary.
4. The transformation function, which is described below, is used to modify the histogram.

(2) $$\begin{array}{l} t\left(r_{k}\right)=\sum_{j=0}^{k}\;p_{r}\left(r_{j}\right) \end{array}$$

(3) $$\begin{array}{l} \text{Where }p_{r}\left(r_{j}\right)=\frac{n_{j}}{n} \end{array}$$

Equations (2) and (3) describe the probability function of input image gray scale value j, *n* is a total number of pixels in input mammogram image, and *n*_*j*_ is input pixel number of gray value j.

5. The adjacent tiles were joined by bilinear interpolation and the image gray scale values were altered according to the revised histograms.

**Figure 5:**
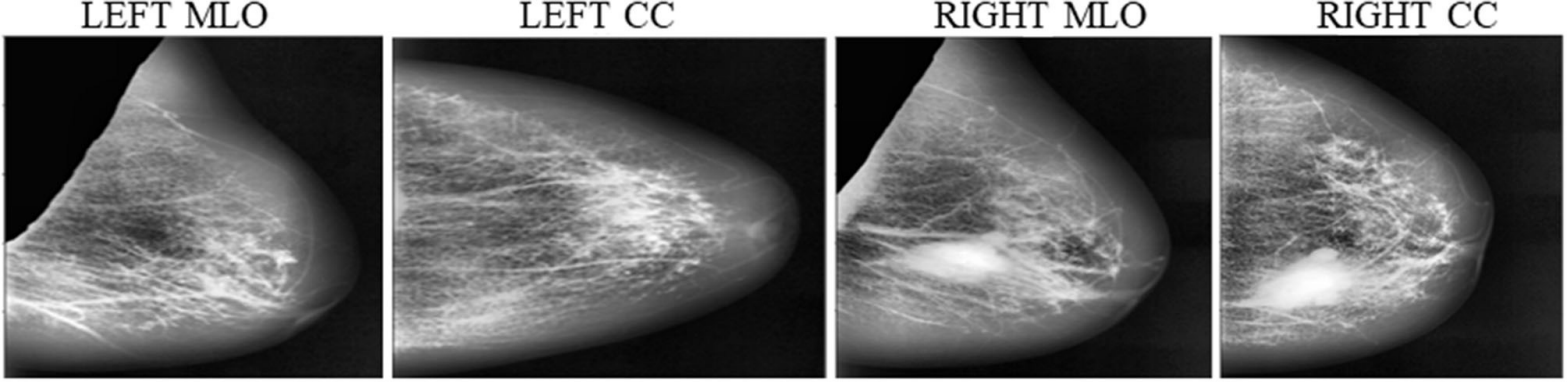
Contrast enhancement of input images.

In this method, the contrast factor is limited to 0.01 to prevent oversaturation of the image, specifically inhomogeneous areas for optimized output. Furthermore, the number of bins for the histogram structure is restricted to 64 over the uniform distribution for contract enhancing transformations. Figure 5 depicts the result of contrast enhancement of the CLAHE algorithm on input images.

### Multichannel Model Development

This article proposes the feature learning ability of multichannel DenseNet architecture presented by Huang et al. (18) toward MBD classification. The proposed method uses four independent DenseNet architecture as four-channel architecture known as multichannel architecture. This architecture is competent in taking all the four views of an individual patient for the classification of MBD.

#### Conversion of Gray Scale Image Into RGB

**Figure 6:**
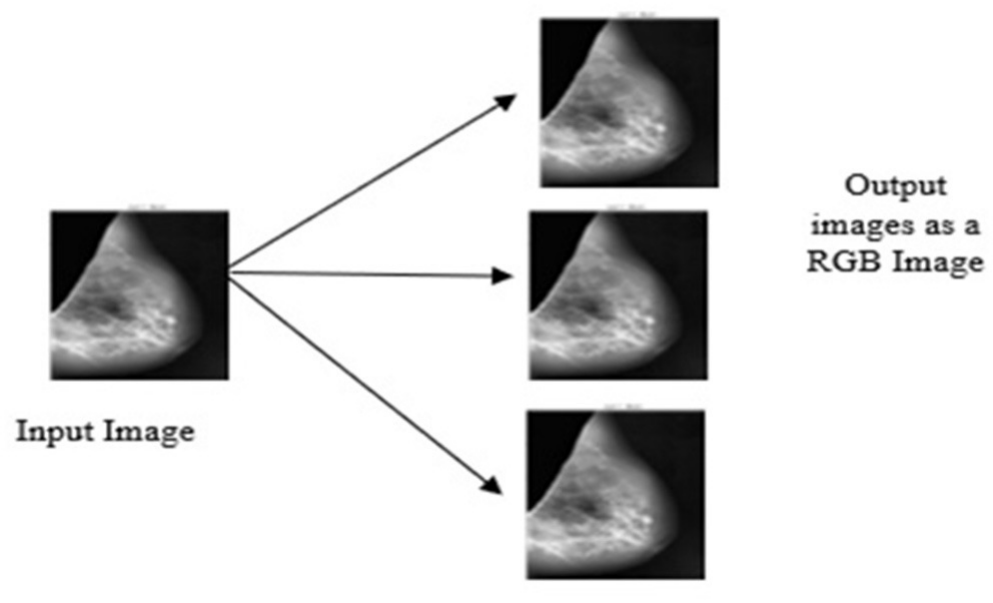
Conversion of grayscale image appears as an RGB.

The DenseNet model is pretrained on red, green and blue channel (RGB) images, but the proposed study uses the gray scale image as the input image. To appear gray scale image as an RGB, perform repetition of image array three times due to which the same image appears on the channels. Then, after duplication of the input image, all the input images are resized into 320 × 320 × 3. Figure 6 depicts the conversion of the gray scale image into a three-channel RGB.

#### Input Convolutional Layer

**Figure 7:**
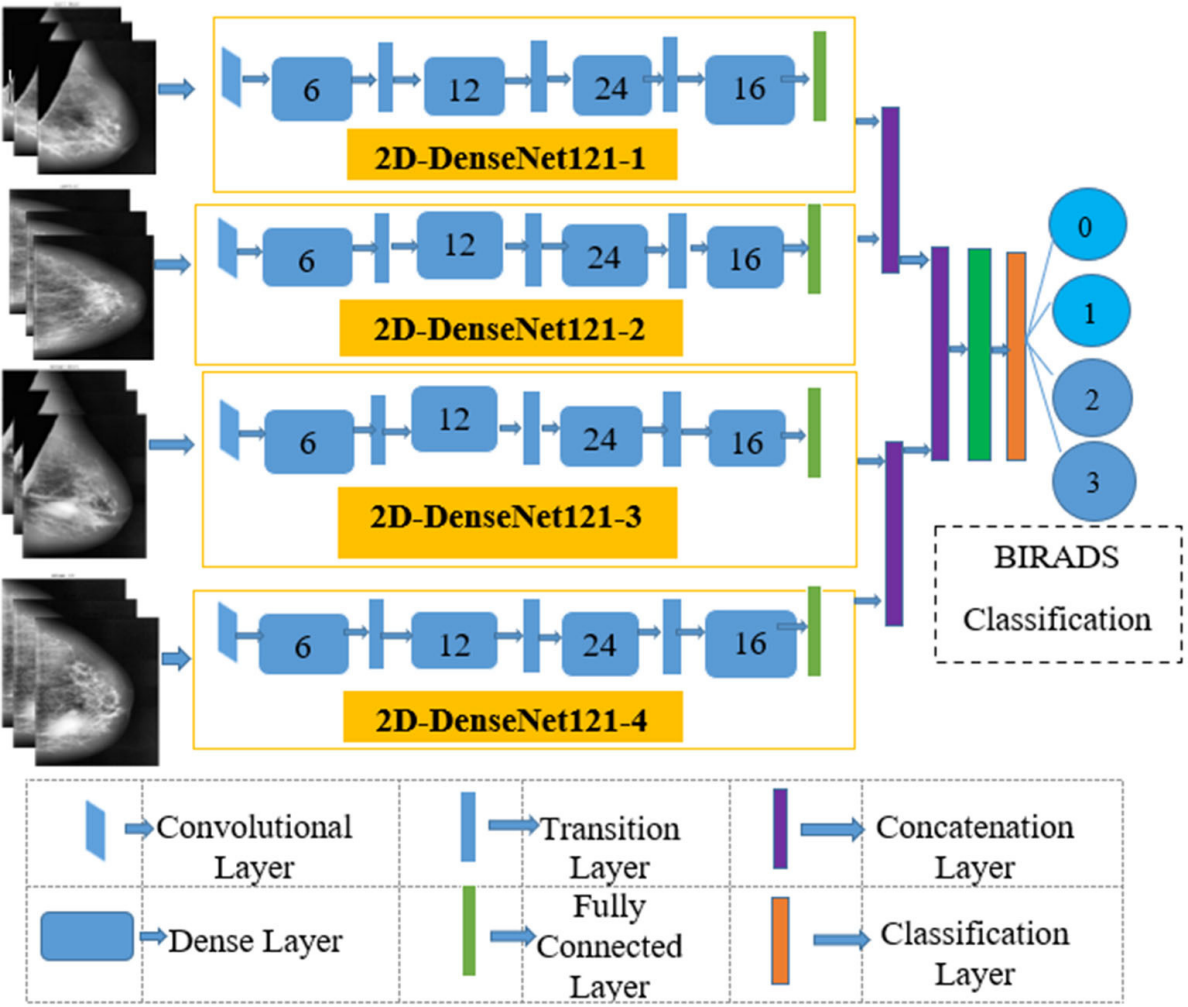
The proposed multichannel Dense-Net Framework for BIRADS classification.

The four input channels of the proposed architecture are marked as L_CC, L_MLO, R_CC, and R_MLO. The fundamental merit of this combination is that all the four views of digital mammography are processed concurrently. Each input layer of DenseNet architecture consists of a convolution layer of the kernel of 7 × 7 with a stride of 2. This convolution operation reduces the input size of the images to 112 × 112 × 3. Input image further passes through a pooling layer of 3 × 3 maximum pooling with stride 2 × 2. Thus, the input layer's convolution and pooling operation reduce the input image size to 56 × 56 × 3 and before passing to the dense blocks. Figure 7 depicts the proposed multichannel architecture.

#### Design of DenseNet Neural Structure

The DenseNet architecture consists of different design variants such as DenseNet121, DenseNet169, DenseNet 201, and DenseNet 264. The DenseNet architecture's fundamental merit is the structure of dense layers precisely designed to take care of downsampling and feature concatenation. Therefore, out of four variants, the proposed architecture uses the DenseNet121 network structure, consisting of a combination of dense block layers and transition layers. Thus, the proposed model uses 58 convolutional layers and a growth rate (*k* = 12), including four dense and two transition layers. In addition, the proposed model consists of comparatively fewer parameters hence, saving computational memory and reducing the overfitting.

##### Dense Block Layer

In four dense blocks, the individual layer is responsible for forming a k-characteristic map after convolution, which also maintains feature maps of each layer are in the same size. K convolution kernels extract all the features from the layers. Parameter k is known as a hyperparameter in DenseNet, which is the growth rate of the network. Each dense layer receives the different inputs from previous layers to reduce computation and enhance the efficiency of the dense block. The dense block internally uses the bottleneck layer (1 × 1 convolution layer between batch normalization, ReLU, and 3 × 3 convolution layer).

##### Transition Layer

This section consists of a batch normalization layer and a one × one convolution layer followed by a two × two average pooling layer. This layer combines two nearby dense block layers to reduce the feature map size. A combination of 4 dense blocks and transition layers converts the image size into 7 × 7 × 3, further provided to the output layer. Each layer connects to the previous stage as an input described by Equation (4):

(4) $$\begin{array}{l} X_{l}=H_{l}\left(\left[x_{0},x_{1},\ldots \ldots ,x_{l-1}\right]\right) \end{array}$$

**Figure 8:**
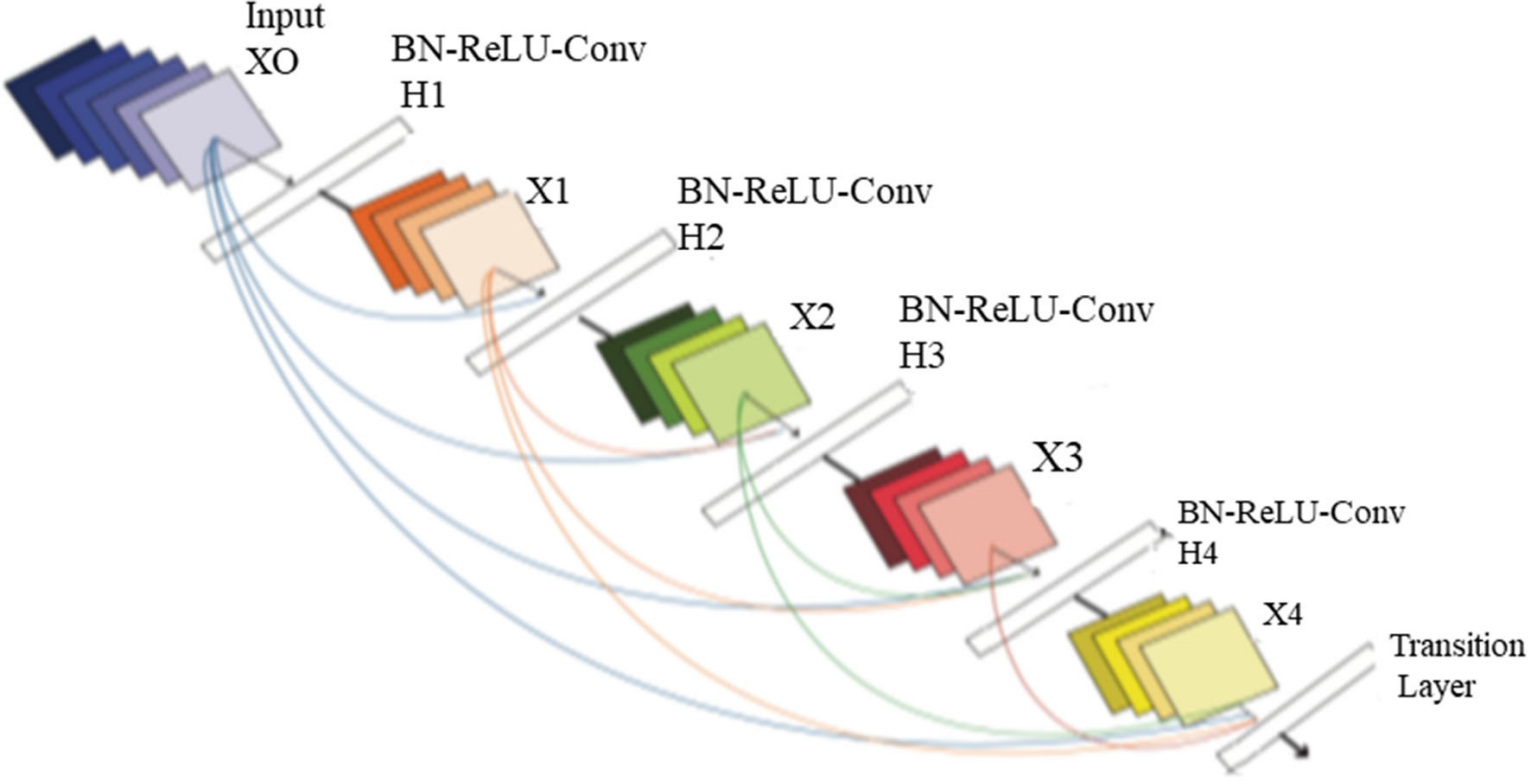
The architecture of dense layer.

A non-linear transformation function *H*_*l*_(.) is responsible for combining series output of batch normalization, ReLU, pooling, and convolution operation. Figure 8 depicts the design architecture of dense layer.

#### Output Classification Layer

The output layer of the proposed architecture consists of a specific average pooling layer for each channel to extract meaningful features. Extracted features are flattened by the flatten layer and are given to the individual dense layer. MBD features received from four-channel are concatenated together with two concatenation blocks. Subsequently, the third concatenation block joins all the proposed method features together. The three dense layers accept all the features collected together and, finally, the classification layer receives the output of three dense layers for classification. The proposed method uses the SoftMax classifier to classify output into four classes as per the BIRADS Density Classification. Table 2 presents the specifications of the proposed method.

**Table 2:** Technical specification of proposed architecture.

**No. of channels**	**04**	
**Layers/Channel**	**Output size/channel**	**Block description/Channel**
Convolution	112 by 112	Kernel 7 × 7 stride 2
Pooling	56 by 56	3 × 3 Max.Pooling, stride 2 × 2
Dense block 1	56 by 56	[1 × 1 Conv] × 6 [3 × 3 Conv] × 6
Transition 1	56 × 56	Batch normalization layer and a 1 × 1 convolution layer followed by 2 × 2 average pooling layer
	28 × 28	
Dense block 2	28 × 28	1 × 1 Conv] × 12 [3 × 3 Conv] × 12
Transition 2	28 × 28	Batch normalization layer and a 1 × 1 convolution layer followed by 2 × 2 average pooling layer
	14 × 14	
Dense block 3	14 × 14	1 × 1 Conv] × 24 [3 × 3 Conv] × 24
Transition layer 3	14 × 14	Batch normalization layer and a 1 × 1 convolution layer followed by 2 × 2 average pooling layer
	7 × 7	
Dense block 4	7 × 7	1 × 1 Conv] × 16 [3 × 3 Conv] × 16
Classification layer	1 × 1	7 × 7 global average pool
	1000D fully connected, SoftMax	

## Analysis of Experiment and Outcomes

**Figure 9:**
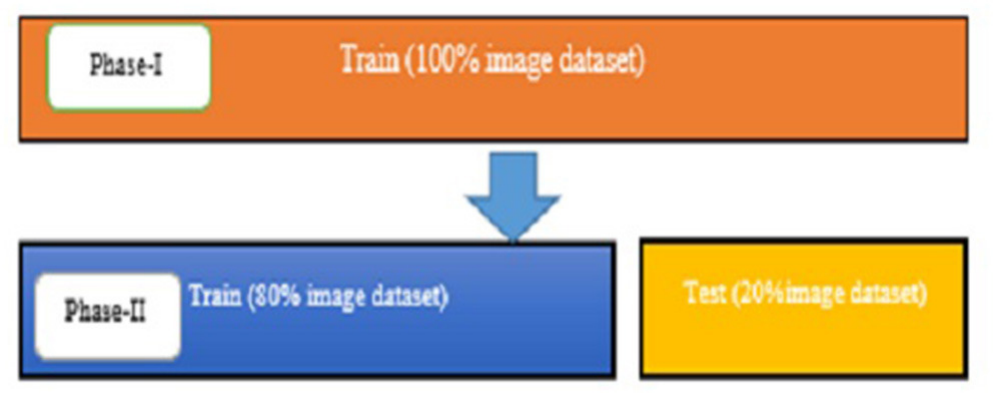
The distribution of image data.

The experimentally proposed design is trained and tested upon the PyTorch framework on Google Colaboratory, a free online cloud-based Jupiter notebook environment. The proposed method input dataset is not sufficient to split into the train, validate, and test data sets; hence, training and testing of the model are done in two phases. Figure 9 depicts the image data distribution during training (phase I) and training and testing (phase II).

### Phase I

The entire model is trained with stochastic gradient descent (SGD) algorithm using batch sizes 4 and 30 epoch on the whole dataset. SGD is an optimization algorithm that estimates the error gradient for the model's current state with an example of a training set; after this, it updates the weights of the model using backpropagation (35–37). Equation (5) describes weight updating mechanism in SGD algorithm.

(5) $$\begin{array}{l} w_{new\;=\;W_{old}\;}-n\nabla Qi\left(w_{new\;}\right) \end{array}$$

Where *w*_*new*_ is new weight, is weight at previous iteration is learning rate and weight gradient. The primary merit of this algorithm is that it updates parameters for each training example and performs one update at one time. Thus, SGD is faster and can also learn online. The weight updating step size is the learning rate of the model. The learning rate is a configurable hyperparameter that controls the speed by which the model learns. The initial learning rate for this model is 0.1 (default value) and further divided by ten at 50 and 75% of the total training epochs. The categorical cross-entropy acts as a loss function in this model, quantifying the difference between four probability distributions. This loss function works well with the SoftMax activation function in multiclass classification. Equation (6) describes the categorical cross-entropy mathematically, which is:

(6) $$\begin{array}{l} C.E.=-\sum_{i}^{c}t_{i}log\left(s_{i}\right) \end{array}$$

Where *C*.*E*. is cross-entropy *t*_*i*_ and *s*_*i*_ ground truth and the convolutional neural network (CNN) score for each class *i* in *c*. Table 3 presents the setting of different hyperparameters used to obtain the optimized results of the proposed architecture.

**Table 3:** Setting of hyperparameters used during experiment.

**Hyper parameters**	**Value**
Model	Multichannel-Dense Net
No. of channel	04
Model initial learning rate	0.1
Image size	320 × 320 × 3
Batch size	04
Target labels	Ground Truth
Data augmentation	Not used
Loss function	Categorical cross-entropy
Optimization algorithm	Stochastic gradient decent
Validation parameter	Classification accuracy

**Figure 10:**
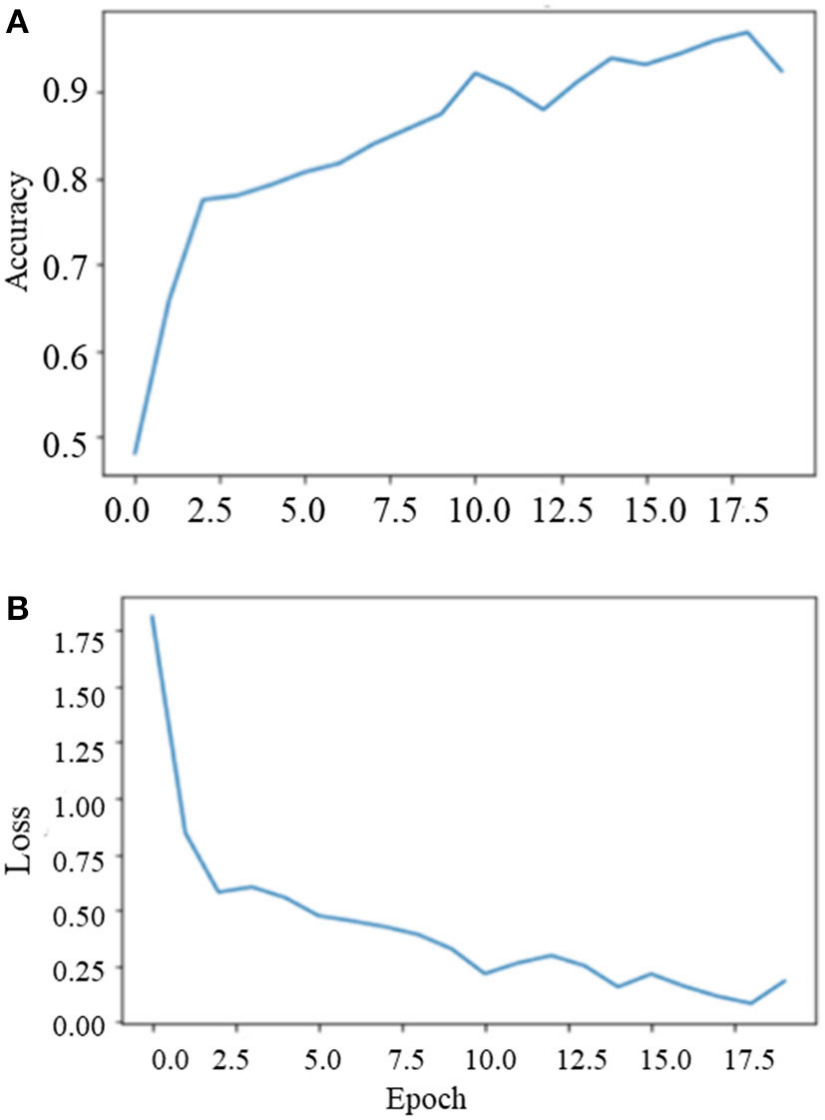
Training phase performance of the model **(A)** model accuracy and **(B)** model loss.

During the training on the entire dataset, the best classification accuracy score was 96.35% at 18 epochs with a loss factor of 0.1344. Figure 10 depicts the training phase results on the dataset as a whole.

### Phase II

**Figure 11:**
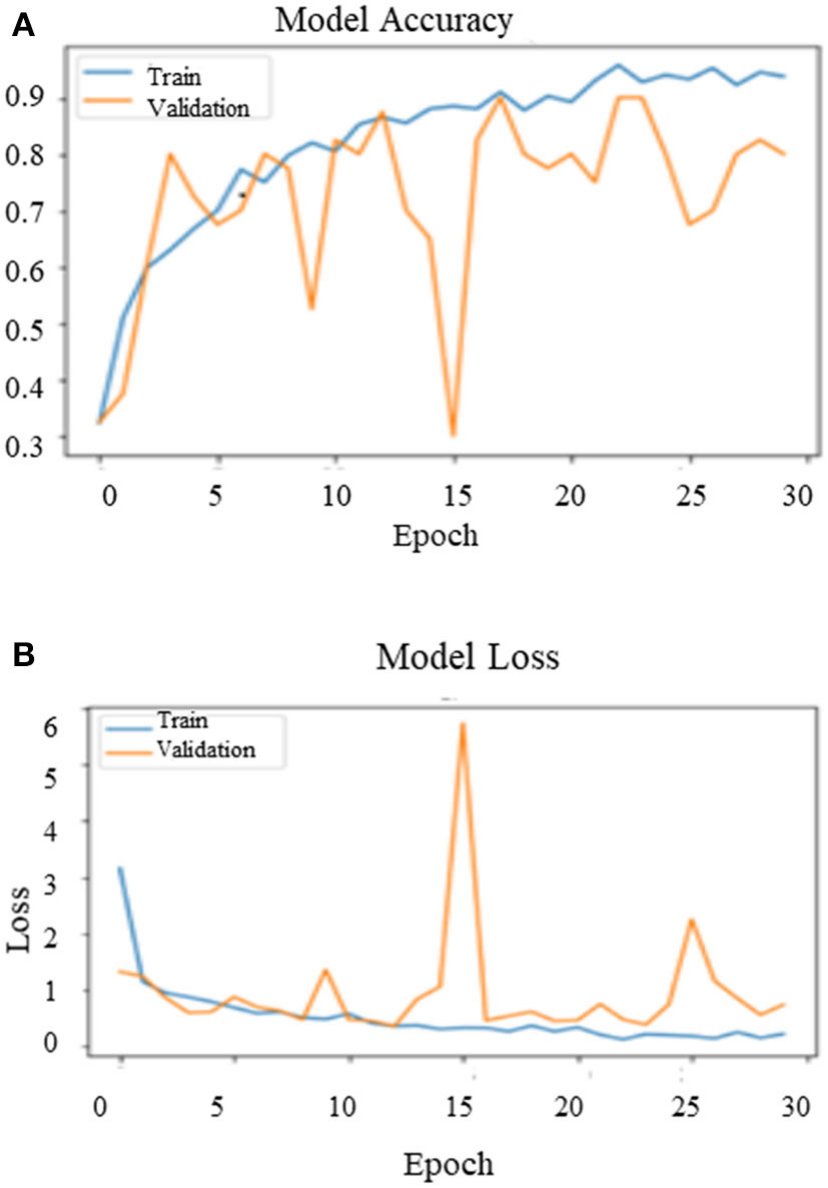
Validation results of the proposed model in phase-II **(A)** model accuracy **(B)** model loss.

After training the model on the entire dataset, the proposed model performance is validated by spitting the image dataset in a ratio of 80% as training and 20% as testing. The proposed model performed significantly well on all the BIRADS density classes during the testing phase and recorded the best classification accuracy, 90.00%, with a validation loss of 0.3814. Figure 11 depict the outcomes of validation over the training model.

### Results Evaluation

**Figure 12:**
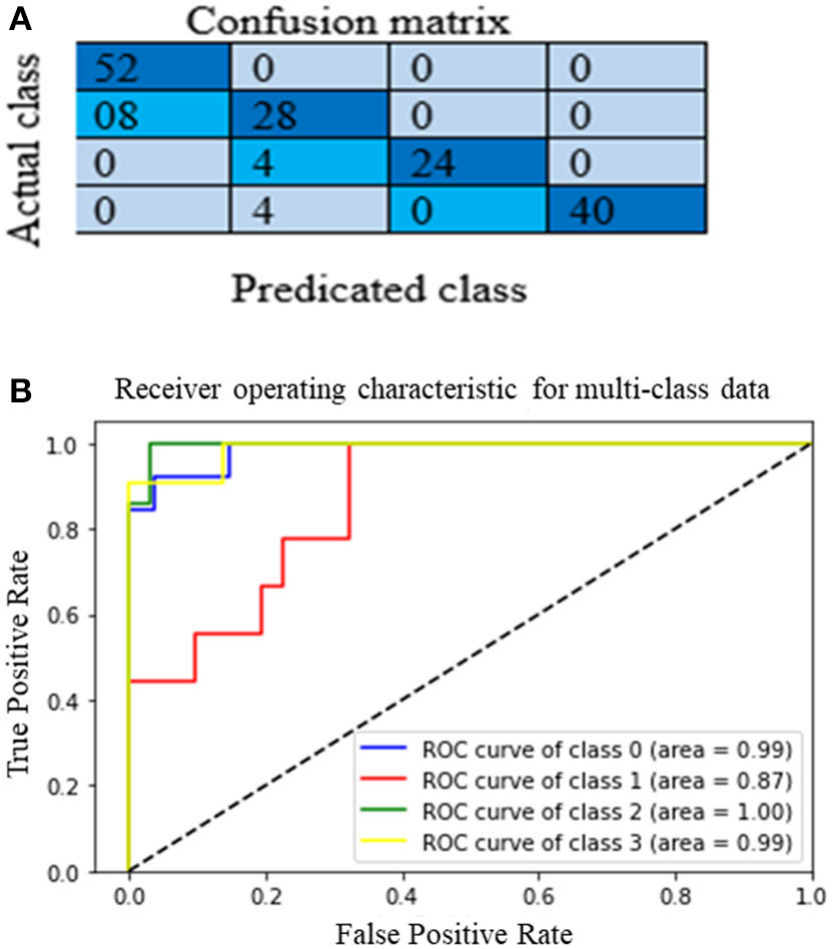
**(A)** The Heat map **(B)** and the ROC curve of the proposed model.

The proposed multichannel DenseNet architecture performance is analyzed from the confusion matrix of the model on the test dataset. Figure 12A shows the proposed architecture's heat map (confusion matrix) on the test dataset. The heat map helps to analyze which category is correctly classified by the proposed architecture. The main diagonal darker version indicates a better classification rate. Although it is clear that the model is working well in classes A, C, and D, there is some confusion in classifying category class B. Still, this model correctly classifies heterogeneous dense (C) and extremely dense (D), which is the essential bottleneck behind MBD classification. Figure 12 depict the heat map and the AUC curve, respectively, of the proposed model.

Evaluation of the classification results of the intended architecture is performed in terms of precision, recall, the F1-score, and classification accuracy. Among those parameters, precision is the proportion of samples with optimistic predictions concerning the total number of correct positive samples. The recall ratio of correctly predicted samples to the whole samples and the F1-score are the precision and recall weight. Finally, classification accuracy is the total correct predictions to the total number of samples. Equations (7) to (10) define precision, recall, the F1-score, and classification accuracy, respectively:

(7) $$\begin{array}{l} Acc=\frac{N_{A}+N_{B}+N_{C}+N_{C}}{N_{All}} \end{array}$$

(8) $$\begin{array}{l} F1-score=\frac{2PR}{P+R} \end{array}$$

Where *N*_*All*_ *is* the total number of images and *Na, Nb, Nc, and Nd* signify the number of images in MBD classes A, B, C, and D.

(9) $$\begin{array}{l} P=\frac{N_{t}}{N_{i}}\;\times 100 \end{array}$$

(10) $$\begin{array}{l} R=\frac{Nt}{Nf}\times \;100 \end{array}$$

Where, Nt is the correct number of predictions of a specific category and Ni is all the number of forecasts of a class and indicates the actual number of the category, among them are precision and recall, respectively. The model accuracy is the ratio of the sum of the diagonal elements to all the elements. Thus, it acts as an indicator of the overall prediction of the model. Table 4 presents the model's overall performance in detail and variation in precision rate, the recall rate, and the F1-score rate under different categories. The number (or percentage) of accurate positive samples for all the four BIRADS density classes are 92, 75.5, 92.2, and 94.7% of their respective totals. From the results shown in Table 4, there is no confusion between classes A and C, B and C, and C and D. The proposed algorithm results are consistent with the results evaluated by the radiologists, which are a positive sign that indicates that deep learning models are helpful for the classification of MBD. Another graphical technique utilized to investigate the realization of computer-aided diagnostic methods is the receiver operating characteristic (ROC), as shown in Figure 12B. This curve analysis performance of the prototype should be in terms of true positive rate (TP) and false positive rate (FP). The AUC value refers to the area enclosed by the ROC curve in the [0, 1] period and the X-axis. The higher the AUC content, the more reliable the realization of the model. It additionally highlights the ability of the model to differentiate among the classes.

**Table 4:** Performance parameter of the proposed method.

**BIRADS density classes**	**Precision**	**Recall**	**F1-score**	**Overall classification accuracy**	**Overall AUC**
Predominantly fatty-class A	1	0.866	0.92	0.9006	0.9625
Fat with some fibro glandular tissue class B	0.77	0.77	0.755		
Heterogeneous dense-class C	0.857	1	0.922		
Extremely dense-class D	0.90	1	0.947		

## Discussion

Inconsistency in MBD assessment is the fundamental reason behind unnecessary extra screening procedures and cause for patient anxiety (41–43). Due to improved system architecture and hardware capability, the deep learning model can be an alternative for medical image classification. Still, the need for a larger dataset and vanishing gradient are the primary bottleneck issues to obtain state-of-the art results from deep learning models (44).

### Advantages of Proposed Method

This study article proposes multichannel DenseNet architecture for MBD classification to investigate the performance of DenseNet architecture on a smaller dataset. The proposed architecture has recorded good classification performance in four-class classification. Furthermore, the visualization results show that the model can distinguish between all the BIRADS density classes, especially in “scattered density” and “heterogeneously dense category of the BIRADS.” Thus, this model can help the radiologists to classify the BIRADS density classes quickly. The main reasons for the excellent performance of this model are as follows:

1. Instead of using raw images, the proposed architecture uses preprocessed images. Hence, there are no high-density areas such as pectoral muscle and tags on mammograms, which are helpful to increase the classification accuracy of the proposed architecture.
2. The proposed method uses a contrast enhancement technique to improve the quality of training data.
3. The model contains four DenseNet branches, which extract the features of mammograms from four views of the single patient, so that the network can focus on a broader range of spatial information.
4. Due to multichannel architecture, it is possible to process all the views of a single patient simultaneously.
5. Subsequently, with multichannel and multiview architecture, it is probable to consolidate all the features collectively; consequently, the performance of the intended model is found more reliable than single-view classification.

### Comparison With Existing Methods

While there are variations in related datasets and evaluation methods, the straightforward comparison is challenging to researchers. This section compiles the proposed algorithm's comparison state with existing classifications. To study the interobserver variation in MBD assessment, N Kaiser et al. (24) proposed the novel multichannel VGG architecture. This approach uses a total of 8,150 digital mammograms, divided into 600 cases. This method recorded 88% two-class classification accuracy (dense and nondense) with the AUC of 0.954. Besides, these results are also compared with the 32 individual radiologist's panel's density ground truth. This study reveals that the deep learning approach performs better than average radiologists. Thus, we only need to refine the deep learning model for MBD classification. However, the fundamental limitation of this method is that the gradient flows from the final layer to the initial layer; hence, vanishing gradient problem takes place, which increases training time and reduces the classification accuracy. In the proposed method, due to DenseNet architecture, all the layers are directly connected in feed-forward nature, acting as an effective solution for vanishing gradient and reducing training time. Thus, the results of the proposed algorithm outperform this method on a smaller dataset.

Another method directly comparable to the proposed method is the optimized lightweight deep learning architecture proposed by Shi et al. (22). The elemental focus of this method is to overcome the requirement of the larger dataset and vanishing gradient problem of the deep learning algorithm. This method combines three CNN layers, one dense layer, and an output layer to classify MBD. This architecture is tested on the 322 mini-MIAS dataset with different data augmentation techniques and recorded 83.6% classification accuracy. However, due to the smaller dataset, this architecture has limitations regarding moderate classification accuracy and low stability of the network. Therefore, the proposed method used the concept of transfer learning and multichannel architecture to overcome these limitations. As a result, the proposed model outperforms this method in classification accuracy on a smaller dataset. Table 5 provides the comparative state of the proposed method with other different existing methods.

**Table 5:** Comparative status of the proposed method with current state-of-the-art methods.

**References**	**Dataset**	**Proposed method**	**Classification accuracy**
Wu et al. (20)	2,00,000	A deep convolutional network with 100 layers.	0.825 on Four views
Ciritsis et al. (19)	20,578	A deep convolutional network with 11 layers and performed analysis separately on CC and MLO views.	0.897 On CC views and 0.866 on MLO views.
Kaiser et al. (24)	8,150	A multichannel architecture with transfer learning by VGG-Net.	0.88 on all four views
Shi et al. (22)	322	A light-weight deep learning architecture with 3 convolutional layers.	0.836 On MLO views.
Deng et al. (36)	18,157	A single channel architecture with transfer learning by Dense Net 121 combined with SE-Attention network.	0.9179 on all Four views
Proposed method	800	A multichannel architecture with transfer learning with Dense Net 121	0.90 on Four views

### Limitations and Future Study

Even though the proposed method has improved the classification performance of the BIRADS density classes, some issues still need to be addressed. First, this study uses a smaller amount of image data and no image enhancement strategies are used to expand the dataset. Hence, model performance, especially stability during validation, is affected due to limited image data and model found it a little confusing to classify classes A and B. Therefore, in future study, data enhancement techniques will improve the model's performance. Second, the proposed study addresses only one type of dataset; hence, this approach does not address the robustness of the model. Future study will address the robustness of the model by training the model with different vendor-specific image datasets and testing results of all the mentioned state-of-the-art methods with the proposed method with the same work environment. The proposed study will be undoubtedly helpful in addressing the issues mentioned above.

## Conclusion

In summary, the primary objective behind this study is to classify MBD as per the BIRADS classification. This study proposes the novel approach of multichannel architecture with DenseNet121 for the objective assessment of MBD. The proposed framework uses the four views of a single patient to enhance feature learning ability through a multiview approach. In the method, image contrast enhancement and preprocessing of the input image are implemented to enhance the condition of the training image data. The input images are processed through multichannel architecture to extract and fuse all the features. Analysis of the results suggests that the proposed model successfully distinguishes between all the BIRADS density classes, but is predominantly found superior in the two most distinctive and challenging BIRADS categories: “BIRADS_C” and “BIRADS_D.” Classification accuracy of the proposed model is recorded at 96.67% during training and 90.06% during testing and the average AUC of 0.9625. The introduced design consists of some weaknesses discussed and will be addressed in future study; with certain modifications, the proposed method is suitable for application in clinical workflow in breast cancer screening to avoid false recalls.

## Data Availability Statement

The original contributions presented in the study are included in the article/supplementary material, further inquiries can be directed to the corresponding author/s.

## Author Contributions

All authors listed have made a substantial, direct, and intellectual contribution to the work and approved it for publication.

## Conflict of Interest

The authors declare that the research was conducted in the absence of any commercial or financial relationships that could be construed as a potential conflict of interest.

## Publisher's Note

All claims expressed in this article are solely those of the authors and do not necessarily represent those of their affiliated organizations, or those of the publisher, the editors and the reviewers. Any product that may be evaluated in this article, or claim that may be made by its manufacturer, is not guaranteed or endorsed by the publisher.
